# COVID-Bot, an Intelligent System for COVID-19 Vaccination Screening: Design and Development

**DOI:** 10.2196/39157

**Published:** 2022-10-27

**Authors:** Chinedu Wilfred Okonkwo, Lateef Babatunde Amusa, Hossana Twinomurinzi

**Affiliations:** 1 Centre for Applied Data Sciences College of Business and Economics University of Johannesburg Johannesburg South Africa

**Keywords:** chatbot, COVID-Bot, COVID-19, students, vaccine, exemption letter, vaccination, artificial intelligence

## Abstract

**Background:**

Coronavirus continues to spread worldwide, causing various health and economic disruptions. One of the most important approaches to controlling the spread of this disease is to use an artificial intelligence (AI)–based technological intervention, such as a chatbot system. Chatbots can aid in the fight against the spread of COVID-19.

**Objective:**

This paper introduces COVID-Bot, an intelligent interactive system that can help screen students and confirm their COVID-19 vaccination status.

**Methods:**

The design and development of COVID-Bot followed the principles of the design science research (DSR) process, which is a research method for creating a new scientific artifact. COVID-Bot was developed and implemented using the SnatchBot chatbot application programming interface (API) and its predefined tools, which are driven by various natural language processing algorithms.

**Results:**

An evaluation was carried out through a survey that involved 106 university students in determining the functionality, compatibility, reliability, and usability of COVID-Bot. The findings indicated that 92 (86.8%) of the participants agreed that the chatbot functions well, 85 (80.2%) agreed that it fits well with their mobile devices and their lifestyle, 86 (81.1%) agreed that it has the potential to produce accurate and consistent responses, and 85 (80.2%) agreed that it is easy to use. The average obtained α was .87, indicating satisfactory reliability.

**Conclusions:**

This study demonstrates that incorporating chatbot technology into the educational system can combat the spread of COVID-19 among university students. The intelligent system does this by interacting with students to determine their vaccination status.

## Introduction

### Background

COVID-19 has spread worldwide, affecting many aspects of daily life, including education. This pandemic has resulted in the deaths of millions of people, job losses, illnesses, physical communication restrictions, and changes in organizational operating methods [1-3]. Various response strategies, including lockdowns, social distancing, wearing of face masks, and vaccination, have been introduced to control the spread of the virus. Scientists have developed several vaccine candidates to help combat the disease, including Pfizer/BioNTech, Moderna mRNA vaccines, the Johnson and Johnson viral vector vaccine, and the AstraZeneca viral vector vaccine [4]. Although these vaccines aid in the fight against disease spread, other strategies are required to reduce coronavirus infection [5,6].

Artificial intelligence (AI) approaches can aid in the dissemination of critical information worldwide, as well as reduce incorrect information concerning COVID-19 [7,8]. AI-powered chatbot systems are the latest technology innovations used to combat the COVID-19 pandemic [9,10]. Chatbots are intelligent conversational agents interacting with users to respond to their questions [11]. Chatbots can be deployed on websites or social networking platforms, such as Facebook, Skype, and WhatsApp [12,13]. For example, the World Health Organization (WHO) technology initiative developed a chatbot to combat COVID-19; this system is accessible via Facebook and WhatsApp [14]. Individuals or users can use the chatbot to get answers to their questions about how to protect themselves from coronavirus, learn more about the disease, and help prevent its spread. A chatbot is highly beneficial because it can offer a single, reliable answer to most user inquiries and delivers brief information from trusted sources, which can be easier than an extensive list of results from online search engines or social media [15].

The existence of coronavirus is no longer in doubt, and people are returning to their everyday lives, while taking necessary precautions to avoid the disease. Students in higher education are returning to schools to begin classroom or face-to-face teaching and learning. Before a student is allowed to enter the school grounds, most institutions conduct COVID-19 screening at their various entrances. This screening is done manually, which causes some inconveniences, such as long lines, time waste, fatigue, and human errors. Furthermore, this process may pose a significant challenge to the authorities or administration, necessitating new technologies and automation. AI technology, such as chatbots, can aid in the screening process.

This study presents COVID-Bot, an AI-powered chatbot that can screen higher education students, determining their receipt of the COVID-19 vaccine. COVID-Bot was built using SnatchBot, a chatbot development platform [11,16]. This research will contribute significantly to the literature on technology acceptability in health care and education systems. First, we developed a chatbot that can verify whether higher education students have received the COVID-19 vaccine. Second, an evaluation of the COVID-Bot showed that the tool is helpful to students and other stakeholders in the process of determining the COVID-19 vaccination status in educational institutions.

The remainder of this paper is structured as follows. The next section presents the background information and related works, followed by the research methodology, design and development of COVID-Bot, implementation, results, and evaluation and application of the system. The final section of the study concludes the study.

### Theoretical Aspects

This section discusses the theoretical aspects of the research, including chatbot technology, the educational impact of the COVID-19 pandemic, and other related works.

#### Chatbot Technology

A chatbot system is an AI-powered technology that can interact with people and provide accurate and immediate answers [17,18]. It is an intelligent agent that can communicate with a user, answer a series of questions, and offer the correct response [19]. Some other names for chatbots are intelligent agents, conversational agents, digital assistants, and clever bots. A chatbot system can be built using a variety of development platforms, including SnatchBot, IBM Watson, Microsoft Azure, and Google [11,20]. Developers can use these frameworks to create chatbot interactions that address a wide range of educational challenges. Chatbots are designed to enhance various working experiences and are a game changer in the inventive era of the Fourth Industrial Revolution (4IR). The introduction of chatbot technology has brought in a lot of new opportunities for a variety of industries [21], including education, health care, and agriculture.

In the education domain, chatbots are not only used to improve students' learning and interaction abilities but also help the teaching staff by introducing automation [21]. Chatbots in education improve connectedness, enhance efficiency, and decrease ambiguity in interactions. They can easily deliver a targeted, individualized, and result-oriented online learning platform [22], which is precisely 1 of the major expectations of current educational institutions. The increased use of technology in daily life impacts how students learn and absorb information. The use of chatbots in education allows for the democratization of education because they do not consider the student's location, resources, or language. Chatbot systems, in general, provide quick and rapid replies, individualized learning experiences, automated tasks, and centralized learning.

However, in the light of the growing difficulties in attending to patients during a health crisis or pandemic, such as COVID-19, the health care sector has shifted its focus to strengthening digital health care services. Health care professionals are using chatbots to assist patients 24 hours a day, 7 days a week, which is a major changer for the business [23,24]. Health care chatbots can deliver accurate, up-to-date information, while improving the patient experience. One of the best ways to manage the COVID-19 crisis is to stay safe and be informed. Accurate information about the pandemic and individuals’ state of health is essential in combating the spread of the disease. Chatbots can help in the war against misinformation and provide guidance to treatment, direction to basic health care facilities, safety precautions, and self-evaluation or screening. As students return to the classroom learning system, it is essential to provide an automatic and interactive agent that can help screen their state of health anytime and anywhere, while observing safety precautions.

#### The Educational Impact of the COVID-19 Pandemic

Millions of people worldwide have died due to the novel coronavirus disease [2]. As a result, some human activities have been forced to cease. Many individuals work under severe constraints, and many others work from home. Higher education was no exception, with most institutions worldwide closing their campuses and replacing conventional learning techniques with online learning using various software tools and applications.

Naturally, this abrupt change generated uncertainty for both administrative personnel and students at most institutions, where many policies had to be revised to reflect the new reality [25,26]. Furthermore, the administrative staff could not respond to the massive rush of inquiries and requests made by students on a variety of themes [26]. According to Igoe and Chadwick [27], the COVID-19 pandemic has posed immediate and long-term difficulties to higher education, notably in administration, financial, academic, technological, and learning possibilities.

- Administration: Since the emergence of COVID-19, higher education administration has struggled to develop appropriate actions and response activities in response to the pandemic. During the pandemic, higher education institutions performed their teaching and learning online [28], which created various administrative issues, such as monitoring/supervision, faculty training for e-learning technologies, and student opposition [29]. Addressing this challenge requires proper leadership and management with clear and defined responsibilities [30].
- Funding: The pandemic has adversely impacted global economic growth, which has affected the funding of educational activities. Because many economies are still recovering from the effects of the COVID-19 pandemic, higher education funding is projected to reduce for a while, especially for public institutions [31].
- Remote learning: Remote learning occurs outside of traditional classroom settings and is frequently aided by digital platforms, such as online classrooms and learning management systems [32]. Although various institutions provide their students with data bundles to connect to the online learning system, network connectivity remains a problem, particularly in rural and less developed areas. Students may face difficulties due to remote learning, such as connection troubles, a lack of information and communication technology (ICT) efficacy, overwhelming online assignments and projects, and an inability to adjust to remote learning [33].
- Use of technology: Online learning approach requires the use of modern technologies, such as AI. Unfortunately, some academics and students are reluctant to accept and use these technological tools. This hesitancy may be due to unfamiliarity, which influences technology acceptance behaviors. In addition, they are not ready for radical changes in the architecture of their educational system [34].

To ensure that teaching and learning activities are not halted, the United Nations Educational, Scientific, and Cultural Organisation (UNESCO) launched the Global Education Coalition (GEC), with an emphasis on the need for free support, tools, or services that help continue educational services during the COVID-19 pandemic [35]. Consequently, various automated solutions that swiftly and accurately answered various requests became necessary during and after the pandemic.

#### Related Works

Although we could not find a chatbot created specifically to screen students for their coronavirus vaccination status, there are some related studies.

Sweidan et al [26] introduced a Student Interactive Assistant Android Application with Chatbot (SIAAAC). This app guides students to obtain various excellent academic services during the COVID-19 pandemic. The chatbot includes a campus map and several alerts, can reply to both Arabic and English queries, and covers a wide variety of academic topics. In Brazil, Roque, Cavalcanti [10] created a chatbot called BotCovid, which provides accurate information about COVID-19 and can answer not less than 600 questions. The University of California, San Francisco, created and deployed a digital chatbot to screen health care workers for COVID-19 [36]. Within the first 2 months of operation, the system performed 270,000 screens. Another study created a conversational bot based on natural language processing (NLP) that acts as a personal virtual doctor for chronic patients, providing free primary health care education, information, and advice [36]. In April 2020, Standard Bank South Africa introduced a chatbot on WhatsApp to inform its clients about its services. The chatbot provides factual information about Standard Bank's reaction to COVID-19, as well as answers to some inquiries and connections to official pandemic information sources [28]. This study is unique that it is designed to determine student vaccination status.

### Gap

Although these chatbots are created for COVID-19 pandemic response purposes, none is for screening students in higher education to evaluate their vaccination status. Because the higher education setting contains many people who may be in close proximity to one another, providing an automated system that can determine COVID-19 vaccination status is deemed necessary. COVID-Bot is designed to address this gap.

### Motivation

The COVID-19 pandemic caused unprecedented disruptions in education, resulting in extended school closures and abrupt changes to routine school operations that impacted education systems worldwide [25]. The development of various COVID-19 vaccine candidates and other safety precautions have aided in controlling the spread of the disease and reducing the consequences. Still, there is no cure for the disease yet. As students return to school to resume classroom teaching and learning, there will be an increase in human-human contact and interactions. Controlling and reducing disease transmission in higher education students require knowledge of their vaccination status. Studies have suggested that a technological resource, such as an AI-based chatbot, may help achieve this need [10,26].

## Methods

### Study Design

This study adheres to the principles of the design science research (DSR) process, which is a research approach with the goal of creating a new experience (artifact) for problem solving [37,38]. The DSR process consists of 6 sequential stages: identification, definition, design and development, implementation, evaluation, and communication. In the Gap section, the research problem was identified and described. This study introduced COVID-Bot, an intelligent interactive system that can be used to screen higher education students to determine whether they have received the COVID-19 vaccination. COVID-Bot was built on the SnatchBot platform's principles, which are mostly drag-and-drop without coding. The SnatchBot framework was chosen because it offers an appealing set of functions and a simple user interface. It can also be used to deploy a chatbot on websites and social networking platforms. The system was implemented on a website and demonstrated to a group of selected students, who were given a thorough explanation of the system's objectives and operation. COVID-Bot was evaluated following ISO/IEC 25023:2016 standards, which establish quality metrics for quantitatively evaluating the quality of system and software products [39]. A questionnaire with 2 sections, including personal profiles and system qualities, was developed and used for data collection. The techniques of purposive and convenience sampling were used. These techniques were used because the system was designed specifically for student use, and during the evaluation, all possible available students were contacted. SPSS 25 statistical software was used to analyze the collected data. The research report and practical application of the chatbot system were used to communicate the study findings to the public.

### Ethical Considerations

The study was approved by the College of Business and Economics Research Ethics Committee (approval no. 2022-069). All participants were duly informed, with a detailed description of the system.

### Design and Development of COVID-Bot

This section describes the design and development of COVID-Bot, as well as a working algorithm sample. COVID-Bot was built using the SnatchBot development platform.

#### The SnatchBot Development Platform

SnatchBot is a chatbot framework with an attractive range of functions and an easy-to-use interface, allowing quick deployment of a chatbot to web applications and social networking applications, such as Facebook Messenger, Skype, SnatchApp, Viber, Slack, and Twilio. It enables developers to create visually attractive bots without coding and with a high level of expertise. SnatchBot offers the user an interface unit, a knowledge unit, a message bank, and an integration unit. The knowledge unit includes the query and artificial intelligence markup language (AIML) interfaces. AIML enables the chatbot to compare the user's input to the predefined messages in the message bank and provide the user with a matched answer. The integration unit can distribute this interaction on the website and social media network.

The channel is another distinguishing feature of SnatchBot. It is a built-in application programming interface (API) that allows chatbots to be deployed on websites and social media platforms. COVID-Bot is implemented on a website through the SnatchBot channel. Students can use smartphones and computers to access COVID-Bot via the website. SnatchBot provides a built-in editor for generating simple or sophisticated dialogues with action buttons and translations. It also allows one to establish a range of *interactions* with the chatbot's activity. Interactions are used to characterize the *subjects* in the SnatchBot platform. Interactions define a chatbot's unique activity, while subjects determine the activity's predefined content, such as messages, audios, videos, and graphs. For instance, if the chatbot is required to introduce itself, an interaction called introduction could be created, with the associated subject being the message/text that defines the chatbot's purposes. There are 2 types of connections in SnatchBot: local and global. Local connections connect to a specific interaction, while global connections connect to all interactions.

**Figure 1:**
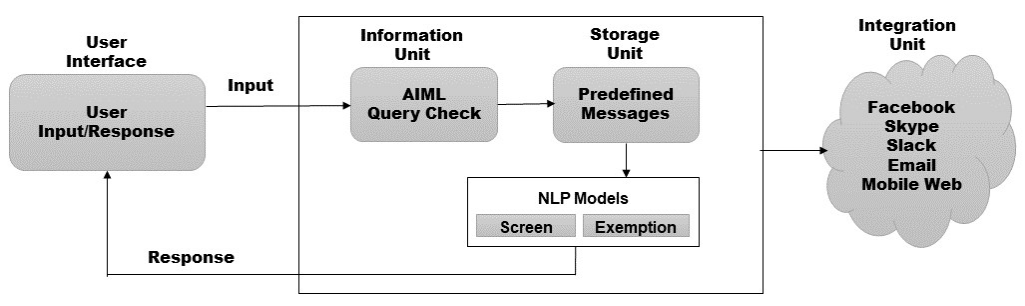
SnatchBot flow diagram. AIML: artificial intelligence markup language; NLP: natural language processing.

Moreover, the SnatchBot platform supports NLP model capabilities. This enables using predefined models and the creation of custom models that can support the chatbot operations. Figure 1 (adapted from Ref. [11]) depicts the SnatchBot operating sequence.

### Design of COVID-Bot

**Figure 2:**
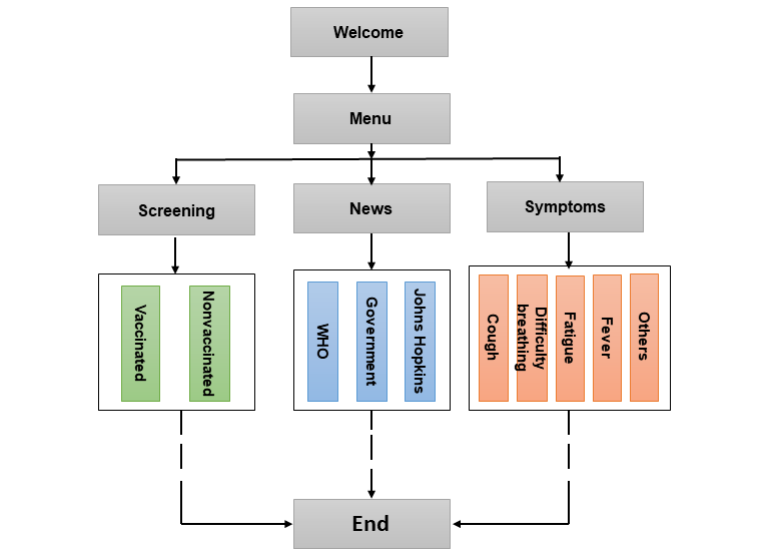
Development scheme of COVID-Bot. WHO: World Health Organization.

COVID-Bot was created using the SnatchBot API and its predefined tools, which are driven by various NLP algorithms. The design of COVID-Bot included a variety of interactions and subjects. Each interaction is given a name, a distinct ID number, and a purpose. The design involves 30 logically and locally connected interactions, including Welcome, Contents, Screen, Exemption, Allowed, and Disallowed. The development scheme of the chatbot is shown in Figure 2.

All connections are expressed as a logical statement in the following form:

If a then b

Else c

The decision flow of COVID-Bot operations is depicted by the following algorithm, adapted from Ref. [8]. The algorithm demonstrates that the user sends a text input, as indicated in line 1, which is compared to the sample in the storage bank or NLP models in line 2 and returns a corresponding response of access granted in line 3 or access denied in line 5 (Textbox 1).

**Decision flow of COVID-Bot operations.**

**Algorithm 1: COVID-Bot Information-Decision**

1. Input: Student Number (user text input);
2. if Student Number Matches Storage Bank then
3. return predefined response (ACCESS GRANTED);
4. else
5. return default answer (ACESS DENIED);

end

The architecture of COVID-Bot consists of 4 main sections:

- Welcome: This section introduces the chatbot and confirms whether the user is ready to converse with the bot.
- Screening: This section checks and confirms whether the user is vaccinated or whether they have been given an exemption letter.
- News: This section provides the latest coronavirus news from the WHO website, the South Africa coronavirus matters website, and the Johns Hopkins University coronavirus information webpage.
- Symptoms: This section presents descriptions of common COVID-19 symptoms.

**Figure 3:**
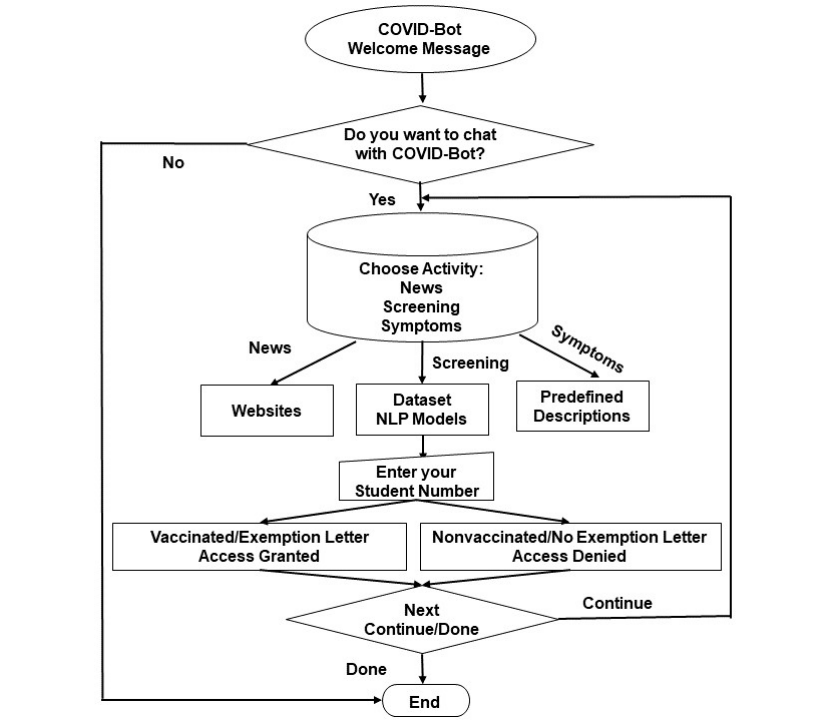
COVID-Bot flow diagram. NLP: natural language processing.

Figure 3 displays an operational decision made by COVID-Bot in response to user inquiries. It demonstrates that when a user (student) connects to COVID-Bot, it delivers a welcome message asking the user whether they wish to interact with the chatbot. If the user responds with yes, COVID-Bot directs them to menu contents, including screening, news, and symptoms. The user chooses the intended activity. During the screening activity, the chatbot requests the student number to verify whether the student has been vaccinated or not vaccinated or has been given an exemption letter to grant or deny them access. If an incorrect student number is entered, the system allows 1 step back to correct it; otherwise, the process ends.

## Results

### Implementation Findings

In this study, we created COVID-Bot, an AI-powered intelligent system that screens higher education students and confirms their COVID-19 vaccination status. COVID-Bot begins its operation by displaying an introduction message to the user and determining whether the user wishes to interact with it. This system serves 3 purposes: (1) It verifies and confirms a user’s COVID-19 vaccination status, (2) it provides the user with access to the most recent coronavirus news from recognized and trusted sources, and (3) it explains COVID-19 symptoms. COVID-Bot requests entry of the student’s information, including name and number. The system takes the student's number and checks to see whether it is in the storage bank. The storage bank contains all the student numbers of those students who have been vaccinated and those who have been granted an exemption. If the student number is found in the storage bank, the student is granted access to the institution's premises and other activities; otherwise, the student is denied access. When a user mistakenly enters an incorrect student number, the system provides them with another chance to correct their mistake. Accordingly, the user must return to the previous option by clicking the back button.

**Figure 4:**
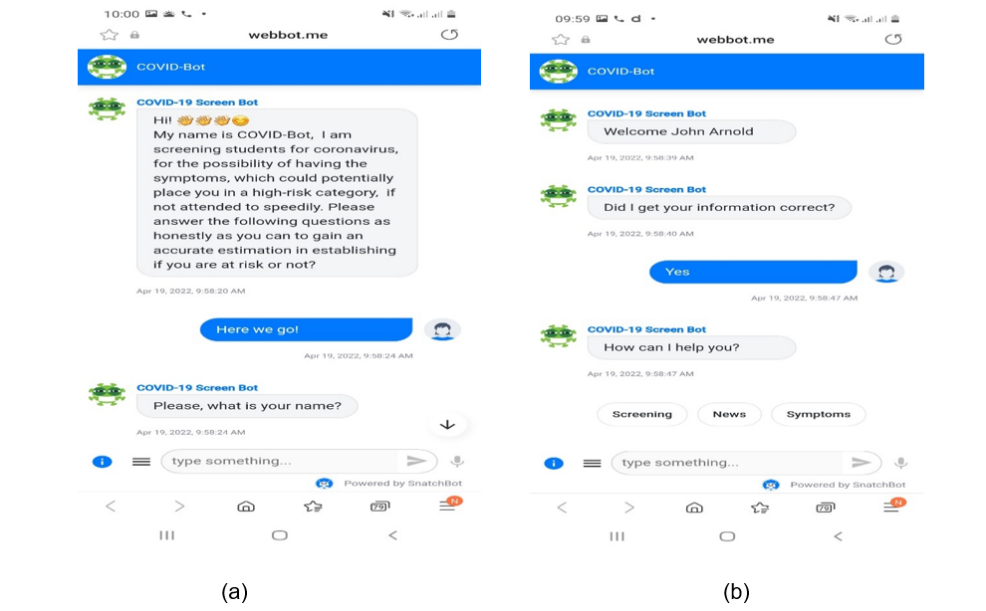
(a) Welcome and (b) activity menus of COVID-Bot.

**Figure 5:**
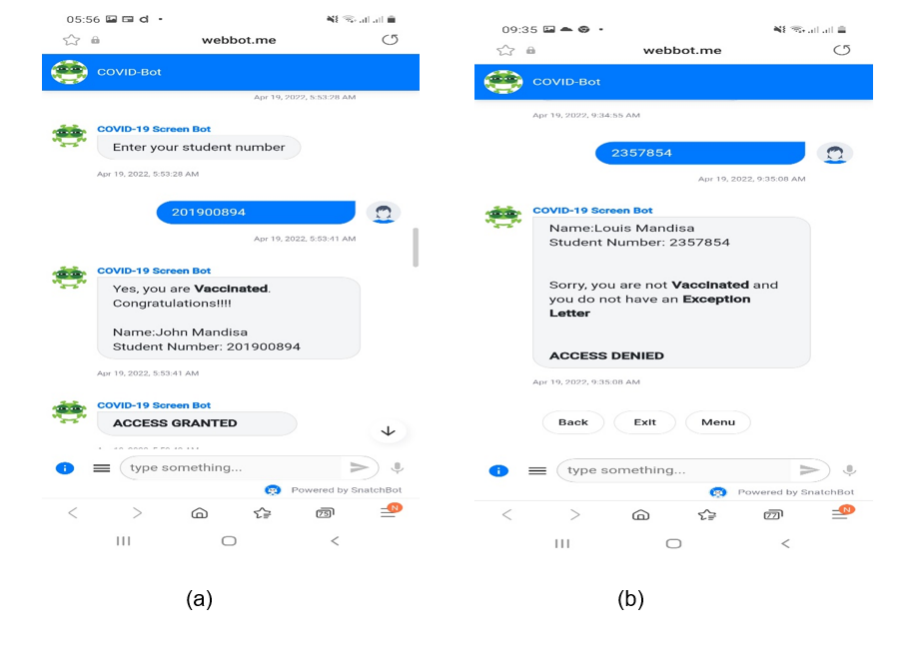
COVID-Bot confirmation message: (a) granting access and (b) denying access to students.

In addition, the COVID-Bot design incorporates 2 NLP models: screen and exemption. These models use machine learning techniques to make decisions based on every input from the user. The models are trained with the samples obtained from data sets of the vaccinated students' list and the nonvaccinated students' list with exemption letters. In other words, the vaccinated data set contains the names and student numbers of all students who have received the COVID-19 vaccine and have a vaccination certificate as evidence. In contrast, the nonvaccinated data set includes the names and student numbers of all students who have not received the COVID-19 vaccine for reasons approved by the institution and have an exemption letter as evidence. The screen model determines and validates the confirmed vaccinated students in the data set, whereas the exemption model determines and validates the confirmed students with exemption letters. COVID-Bot was demonstrated to a group of selected students, who were given an adequate explanation of its objectives and operation. Figures 4a and 4b show the welcome and input activities, and Figures 5a and 5b show the confirmation responses from COVID-Bot.

### Evaluation and Application of COVID-Bot

#### Evaluation

The evaluation of COVID-Bot adhered to the ISO/IEC 25023:2016 standard, which establishes quality metrics for quantitatively evaluating the quality of system and software products [39]. It examines the following user characteristics: functionality, efficiency, compatibility, usability, reliability, maintainability, portability, and security. In this evaluation, students were asked about their perceptions of COVID-Bot’s functionality, compatibility, reliability, and usability. A total of 106 students from the University of Johannesburg were chosen to participate in the study, including those who own smartphones and excluding those who do not. This audience was selected because they are potential users of the system and are actively registered students. Their student numbers were used to train the NLP models. The participants were shown a brief demonstration of the system. An online survey was conducted for quantitative analysis; participants were sent links to the system and a Google form questionnaire to collect their personal information (age, gender, and education) as well as their opinions on the chatbot's qualities. A 5-point Likert scale ranging from 1 (strongly disagree) to 5 (strongly agree) was used for measurement.

#### Reliability Analysis

To determine the reliability of the measuring items, a reliability analysis was performed to obtain Cronbach α coefficients of the measuring constructs. The average obtained α was .87, reflecting satisfactory reliability. Furthermore, a similar correlation value (*r*=0.99) was observed, indicating that the items had a strong relationship. The obtained values of r and α were possible because the evaluation was conducted in a particular context (a university institution) where the participants have similar perceptions. Table 1 shows the results of the reliability analysis.

**Table 1:** Reliability analysis.

Measuring items	Cronbach α
Functionality	.88
Compatibility	.86
Reliability	.85
Usability	.85

### Participant Profile

The sample population consisted of male (n=61, 57.5%) and female (n=45, 42.5%) students at different levels of studies, including undergraduates and postgraduates within the age range of 15-35 years. The predominant age group of undergraduates was 15-25 years (n=83, 78.3%), while postgraduates were 25-35 years old (n=23, 21.7%). In addition, 95 (89.6%) participants agreed they have good knowledge of chatbot technology, while 11 (10.4%) reported having limited knowledge.

Students were asked about their opinions on the following qualities of COVID-Bot: functionality, compatibility, reliability, and usability.

#### Functionality

This test was performed to determine whether the system is functioning correctly to achieve the desired results. Of the 106 participants, 92 (86.8%) agreed or strongly agreed that the system works well and serves its purpose, 10 (9.4%) disagreed, maybe due to their limited knowledge of emerging technologies such as the Chatbot system, and 4 (3.8%) were neutral.

#### Compatibility

This was evaluated to see whether the system will be compatible with the students' existing lifestyle, social norms, and mobile devices. Of the 106 participants, 85 (80.2%) agreed or strongly agreed that the system works well with their smartphones and fits well with their lifestyle, 10 (9.4%) disagreed, possibly due to their ability to use modern technological innovations, and 11 (10.4%) were indifferent.

#### Reliability

This was considered to determine the consistency and accuracy of the system's responses to the user's input or queries. Of the 106 participants, 86 (81.1%) agreed that the system is able to generate appropriate information about vaccinated and nonvaccinated students, as well as those with exemption, 10 (9.4%) disagreed, and 10 (9.4%) were neutral.

#### Usability

To assess the ease with which COVID-Bot can be used to achieve the expected task, usability was evaluated. Of the 106 participants, 85 (80.2%) agreed or strongly agreed that they found the system easy to use, 8 (7.5%) disagreed that they had to repeat the process several times before getting an accurate response due to typos, and 13 (12.3%) were indifferent.

## Discussion

### Principal Findings

COVID-Bot is a conversational agent created to interact with students to confirm whether they are vaccinated or have obtained an exemption letter before being granted access to the institution's premises. This system also provides updated information about the COVID-19 pandemic from recognized sources and descriptions of the symptoms. Chatbots are already being used to battle COVID-19. They have helped eliminate disinformation, aided symptom diagnosis, motivated actions that restrict infection, and reduced mental health costs of the pandemic response [40]. The COVID-19 pandemic is a global issue that can affect anyone regardless of profession or social status. As a result, it is critical to develop a solution that can help reduce the spread of the disease and lessen its societal impact. COVID-Bot was created with this viewpoint in mind.

The evaluation began with a demonstration of the chatbot system to the participants, followed by requesting them to fill out an online questionnaire. According to the questionnaire responses, 86.8% of the participants agreed that the system works well. This suggests the ability of COVID-Bot to determine students' vaccination status and detect those who have received an exemption letter based on the predefined data set. In terms of compatibility, the students were able to use the chatbot with their smartphones, laptops, and desktop computers at the appropriate location and time. As a result, 80.2% of the participants agreed that it is compatible with their lifestyle and activities. The system accuracy and consistency (reliability) test revealed that COVID-Bot is reliable, with 81.1% of the participants agreeing on this factor. This implies that the students' interaction with COVID-Bot produced accurate results. They inquired about their vaccination status, and the system responded with appropriate information. Regarding usability, 80.2% of the participants agreed that the system is easy to use. This means that the design of COVID-Bot is simple enough to use without any prior training or supervision.

Overall, COVID-Bot is designed in a simple manner to accommodate students' use of mobile apps, and it can provide accurate responses to questions about their vaccination status.

### Applications of COVID-Bot

The development of COVID-19 vaccine candidates aids in the fight against the spread of coronavirus. Everyone is expected to get the vaccine, especially those who work in organizations with frequent physical contact with different people, such as academic institutions. As a result, COVID-19 vaccination is required in some institutions. In theory, this study presented the design and development of an intelligent system for COVID-19–related issues, and there are few existing studies in this area. In practice, COVID-Bot can help detect students' COVID-19 vaccination status, thereby helping in the fight against the spread of the deadly coronavirus. Furthermore, it will aid in the safety of students during teaching and learning activities while they are in the institution's environment.

### Comparison With Prior Works

COVID-Bot operations differ from previous related works. It interacts with students intelligently, using their student numbers as input to determine and validate their vaccination status or exemption letter to grant or deny them access to the institution.

### Limitations and Future Works

During the system's implementation, we encountered some limitations in gaining access to students' data sets regarding COVID-19 vaccination status and user privacy concerns. The system is only for students. The entire staff of the institution can be added to future works. It can also be expanded to perform additional functions, such as screening registered and nonregistered students and providing students with financial clearance.

### Conclusion

In this study, COVID-Bot, an intelligent interactive system that can converse with students to determine their COVID-19 vaccination status and confirm whether they can be granted access to the institution and its various activities, was created. COVID-Bot was developed using the SnatchBot chatbot API and can be installed on websites and social networking platforms. An online survey was conducted to evaluate the system. The results indicated that COVID-Bot has good qualities and can be useful in fighting against the spread and impact of COVID-19.

## Abbreviations

AI: artificial intelligence
AIML: artificial intelligence markup language
API: application programming interface
DSR: design science research
NLP: natural language processing
WHO: World Health Organization
